# Exploring association between gastrointestinal heat retention syndrome and recurrent respiratory tract infections in children: a prospective cohort study

**DOI:** 10.1186/s12906-016-1062-8

**Published:** 2016-02-27

**Authors:** Fei Dong, He Yu, Jiaju Ma, Liqun Wu, Tiegang Liu, Guokai Lv, Jianhua Zhen, Xiaofei Li, George Lewith, Xiaohong Gu

**Affiliations:** School of Basic Medical Science, Beijing University of Chinese Medicine, Beijing, China; Beijing Hospital of Traditional Chinese Medicine, Beijing, China; Dongfang Hospital Affiliated to Beijing University of Chinese Medicine, Beijing, China; Beilun Hospital of Chinese Medicine, Ningbo City, China; Complementary and Integrated Medicine Research Unit, Primary Care and Population Sciences, Southampton University, Southampton, UK

**Keywords:** Gastrointestinal heat retention syndrome, Recurrent respiratory tract infection, Children, Prospective cohort study

## Abstract

**Background:**

Recurrent respiratory tract infections (RRTIs) have a negative impact on both children’s health and family wellbeing. Deficiency of ZhengQi used to be an instinct factor driving RRTI in Traditional Chinese Medicine (TCM). Our clinical observations suggest that children with gastrointestinal heat retention syndrome (GHRS) may have a greater risk of catching respiratory tract infections (RTIs). GHRS is a new predisposing factor for RRTI and it is dietary related. This study is aimed to explore association between GHRS and RRTI.

**Methods:**

A prospective cohort study has been conducted in Beijing, China; children aged 1–18 were enrolled. TCM symptoms, demographic and physiological characteristics were recorded by using semi-structured questionnaire. GHRS was considered as a predisposing factor. Children were followed up for next 12 months. We contacted with their parents using a face-to-face questionnaire survey, via email or phone every 3 months. Episodes of RTIs were recorded in detail.

**Results:**

Three hundred thirty four children were enrolled and 307 (91.92 %) followed up for 12 months. The incidence of RTI was 4.32 episodes per child-year (95 % CI 4.03–4.61). 69 (43.13 %) children in the group with GHRS suffered from RRTI; there were 48 (32.65 %) children in group without GHRS. The risk ratio (RR) value of RRTI occurrence was 1.32 (95 % CI 0.91–1.91, *P* = 0.139), and the attributable risk percent (AR%) was 24.28 %. Dry stool and irritability were positively correlated with RTI episodes, age and BMI were negatively correlated with RTI episodes in a linear regression model. Dry stool (OR = 1.510) was positively correlated with RRTI occurrence, age (OR = 0.889) and BMI (OR = 0.858) were negatively correlated with RRTI occurrence in our logistic regression model.

**Conclusions:**

GHRS is associated with RRTI in this cohort. Dry stool was positively associated with RRTI, and BMI was negatively associated with RRTI. Studies with larger sample size and longer follow up are needed to further evaluate this association. Relieving GHRS should be considered when TCM practitioners treat RRTI children, and this may protect children from suffering RTIs.

**Trial registration:**

Chinese Clinical Trial Registry Number: ChiCTR-CCH-13003756

## Background

Respiratory tract infection (RTI) is a major cause of morbidity and mortality worldwide especially in low- and middle-income countries. RTIs are common in children and significantly contribute to pediatric morbidity and mortality [1]. Lower respiratory tract infections (LRTI) (pneumonia predominantly) is one of the leading causes of death in infants and children, especially in developing countries. Approximately 2 million children who die from acute respiratory tract infections (ARTIs) each year [2, 3]. ARTI is the most prevalent in children in primary care: 50 % of children aged 0–4 are diagnosed with ARTI and 10 % of those aged 5–9 [4]. The true incidence of the condition in community may be much higher as usually parents do not consult their doctors when their children develop an upper respiratory tract infection (URTI) [4].

Recurrent respiratory tract infection (RRTI) is a common disease with a higher morbidity both in winter and spring, especially for children [5]. Data suggested 30 % RTIs children suffer from RRTIs, and the morbidity from RRTI in children is increasing [6]. Wenjie Xu and Weihong Liu surveyed children aged from 3 to 6 years in Chaoyang District and Pinggu District of Beijing, China, and found that the morbidity of RRTIs in these regions were 17.8 and 18.7 % [7, 8]. RRTIs impact on children’s health and present significant problems for their families. The economic and social impacts of these infections constitute important challenges to public health because of treatment costs hospitalization, school absenteeism, and loss of working days by parents and caregivers [9].

Many studies have explored the causes of RRTI in children. Generally speaking, recurrent or persistent respiratory infection is suggestive of a deficiency in local or systemic host defenses or an underlying pulmonary disorder which may result from structural, functional or environmental causes [10]. This may include malnutrition such as Vitamin A or serum iron and zinc deficiencies, environmental pollution with heavy metals and an allergic history as well as maternal health during pregnancy [7, 11, 12]. Immune status also impacts on children’s resistance to pathogenic microorganisms [12].

RRTI is deemed to result from congenital and acquired factors, such as deficiency of kidney Qi, lack of proper postnatal care, hypofunctioning of spleen and stomach from the point of view of Traditional Chinese Medicine (TCM) [6]. People with lung, spleen and kidney deficiency are liable to cause exogenous pathogens, thus have more risk to suffer from RRTI. In 2008, there are four different kinds of TCM deficiency syndromes which are declared as the classification of syndromes for the infantile RRTI [5]. However, the constitution and clinical characteristics of children in China have changed along with the life quality and environment. Therefore, we wished to consider new TCM models to explain the pathogenesis of RRTI besides of deficiency of ZhengQi [13–16].

Research work from our team identified that improper diet or eating habits could injure people’s health [13]. This may present as dry mouth, foul breathing, epistaxis, gingivitis, aphthous ulcers, a feverish feeling in the palms and soles, intolerance to heat and sweating. TCM theory reveals that an unhealthy diet which is rich in sugar, protein, energy, salt or additives will do harm to children’ health, while that may be easier to be accessible than before, and they are difficult to digest completely by children, finally end up with dyspepsia as well as the symptoms as above [17]. We defined all these symptoms as gastrointestinal heat retention syndrome (GHRS) based on TCM syndrome differentiation.

GHRS is a syndrome that is associated with increased gastrointestinal heat caused by a metabolic block in energy [17]. Symptoms are involved in including intolerance to heat, excess sweating, dry mouth, a preference for cold drinks desire, foul breath, swift digestion with increased apatite, excessive eye gum, nasal scabs, epistaxis, gingivitis, aphthous ulcers, skin rash, a feverish feeling in the palms and soles, irritability, sleep-talking, bruxism, yellow urine, dry stool, constipation, smelly stools and a reddened tongue with yellow fur. A GHRS diagnosis scale is used to identify people with or without GHRS [18].

Studies have shown that the incidence of functional constipation is 5–10 % in pediatric outpatient clinic [19]. Infantile constipation mostly results from excessive water absorption by the colon, which may be influenced by poor nutrition [20]. Functional constipation in children is often accompanied by internal heat caused by indigestion [21]. The lung and the large intestine are interior-exteriorly related from the point of view of TCM [22]. Studies showed that patients with severe intestinal dysfunction were vulnerable to acute respiratory infections, while facilitating excretion of feces may help to achieve a better outcome for RTIs associated with constipation [23–26]. Studies have revealed that lung and stomach have been closely related, and GHRS could impact the lung function whether with or without RRTI [25].

Previous clinical observations suggested that children with GHRS might have a greater risk of catching RTIs, and experiment studies showed that mice with GHRS were more severely affected after catching FM1 virus infection than the control group from our team work [27]. Our primary aim was to explore the association between GHRS and RRTI in children to identify strategies that could improve patient outcome and prevent RRTI. This study also aimed to supplement the TCM etiology that suggests deficiency of ZhengQi is an instinct factor driving RRTI. We also aimed to describe the morbidity of RRTI in children and explore which disease-related and socio-demographic factors were associated.

## Methods

### Design of study

This is a prospective pragmatic cohort study in secondary care. Participants were enrolled into group with GHRS and group without GHRS according to whether they had GHRS or not, which was used as the ‘exposure factor’. Symptoms of GHRS, disease-related and socio-demographic factors were recorded by using semi-structured questionnaire. We contacted with participants’ parents using face-to-face questionnaire survey, via email or phone every 3 months. The incidence of RTI was recorded over the next 12 months to investigate the association between GHRS and RRTI.

We calculated the sample size with α (significance level) = 0.05, Power (1-β) = 0.90 and P (overall event rate) = 0.8 by the formula as followed [28], N = (Zα + Zβ)^2^ × P(1 – P)/δ^2^ = (1.63 + 1.28)^2^ × 0.8 × 0.2/0.12 = 136.4224 ≈ 136. And 150 participants for each group were planned to recruited with 10 % lost-follow-up concerning.

### Site identification

This study was conducted at several settings in Beijing, China. The participants were recruited from pediatric clinic department of Beijing Dongfang Hospital, pediatric clinic department of Beijing Dongzhimen Hospital, and Guoyitang TCM clinic department of Beijing University of Chinese Medicine.

### Patient recruitment

We consecutively recruited children according to selection criteria. Children who were eligible for inclusion in our study were those with age ≥1 year old and ≤18 years old and with a history of 3 or more RTI episodes in the past 12 months. Fully informed consent was obtained prior to entry. All participants guaranteed to provide the information needed for the case report form (CRF) and be followed up in the next 12 months.

We excluded children with severe circulatory diseases, nervous diseases, urinary diseases, hematological disease. And we also excluded participants with serious digestive system diseases such as severe gastritis, peptic ulcers, colitis, and those who were involved in other clinical trials.

### Ethics

Research approval was obtained from the Ethics Committee of the Beijing University of Chinese Medicine in March 9^th^, 2012. Written informed consents were given to the parents or other guardians of children participants with well explained by researchers before study.

### Data collection

Standard operating procedures (SOPs) training was given before recruiting to all study personnel as quality control, including sticking to inclusion and exclusion criteria, completing the questionnaires, ensuring the quality of CRFs and discussing strategies for keeping participants compliant. Plain language was used and recruitment strategies were standardized while collecting data; and all the information involved was recorded in CRFs.

The GHRS diagnosis scale was used to allocate the participants to GHRS group or non-GHRS group, which has been developed by using mixed methods including literature analysis, expert consensus and Delphi methodologies [18]. In this cohort study, we distinguished whether the participant with or without GHRS by detecting the symptoms of the latest three months to avoid the recall bias and these data was retrospective. In addition, the unified questionnaires were used in face-to-face interview for data collecting and the researchers have been trained with SOPs. Two researchers with TCM background scored the participants separately, and discussed each case until reaching agreement.

After recruitment, data were collected: (1) demographic characteristics: gender, age, ethnic group, education and career of their parents, home address and contacts (phone number and e-mail address); (2) physiological characteristics: height, weight, body max index (BMI); (3) past medical history: RTI episodes in the past 12 months, allergic history, influenza vaccination history, pneumonia vaccination history, and other medical history; (4) TCM symptoms: main symptoms, feeling of coldness or fever, sweating, sleep quality, diet and appetite, defecating and urinating, body examination through inspecting, listening and palpatory examination, tongue and pulse diagnosis; (5) personal life history: temperament characteristics, eating habits, food preference. We collected data at the break of participants suffering RTIs for RRTI patients, or at least 7 days later all the RTI symptoms disappeared.

We contacted their parents using face-to-face questionnaire survey, via email or phone every 3 months. We collected data which included (1) episodes of RTI during this period of time in very detail, such as clinical diagnosis, TCM diagnosis, laboratory tests, treating, disease course; (2) TCM symptoms; (3) eating habits. All participants were followed-up for next 12 months, and at the end of follow-up we recorded participants’ weight and height again. All the information was provided by the close relatives who took care of the children, most of them were mothers of the participants.

An episode of RTI was defined as a child who had been diagnosed with an acute upper respiratory infection (AURI), bronchitis, or pneumonia by a pediatrician [29]. Episode of RRTI was defined as outcome event. RRTI is characterized by repeated occurrence of RTI within relative period of normalcy in patients. The diagnostic criteria of RRTI were based on the consensus guidelines published by the Chinese Medical Association Pediatrics Branch Breathing Group and the Chinese Journal of Pediatrics editorial board in December 2007 [5] (see Table 1).

**Table 1:** Recurrent respiratory infection diagnosis items

Age (years old)	Recurrent respiratory infections (times/year)	Recurrent bronchitis (times/year)	Recurrent pneumonia (times/year)
0–2	7	3	2
3–5	6	2	2
6–14	5	2	2

### Data management and analysis

Data management and analysis was performed by using EPI version 3.1 and SAS version 9.3 for Windows. We contacted participants if anything unclear appearing in CRFs. Two researchers input data separately and Epidata was used which was qualified to check data consistency automatically. We compared participants’ characteristics and discrete clinical signs of children between GHRS group and none GHRS group by using Pearson Chi-square test, or by using Fisher’s exact test if the frequencies were small. Wilcoxon rank sum test was used to compare continuous variables between groups. Descriptive statistics such as frequencies or proportions were completed and presented in tables. The morbidity of RTI in overall cohort was calculated, and the morbidity of URTI and LRTI were calculated separately. Relative risk (RR) of RRTIs with corresponding 95 % confidence intervals (95 % CI) were used as the primary outcome. The independent variables with P value less than 0.2 during bivariate analysis were selected as the factors for multivariable analysis. Stratified analysis was used based on the different disease and models and we investigated significant and independent predictors of RTIs identified in each level. Multivariable linear regressions were fitted by using a step-by-step elimination technique (α_in_ = 0.05, α_out_ = 0.10) to indentify independent predictors separately. Multivariable binary logistic regressions were fitted by using retrospective elimination techniques (α_in_ = 0.10, α_out_ = 0.15) to indentify independent predictors separately. Association between dependent and independent variables was assessed by using odds ratios (OR) value and 95 % CI. Statistical association was considered significant if the P value was less than 0.05.

## Results

### Characteristics of the participants

**Fig. 1:**
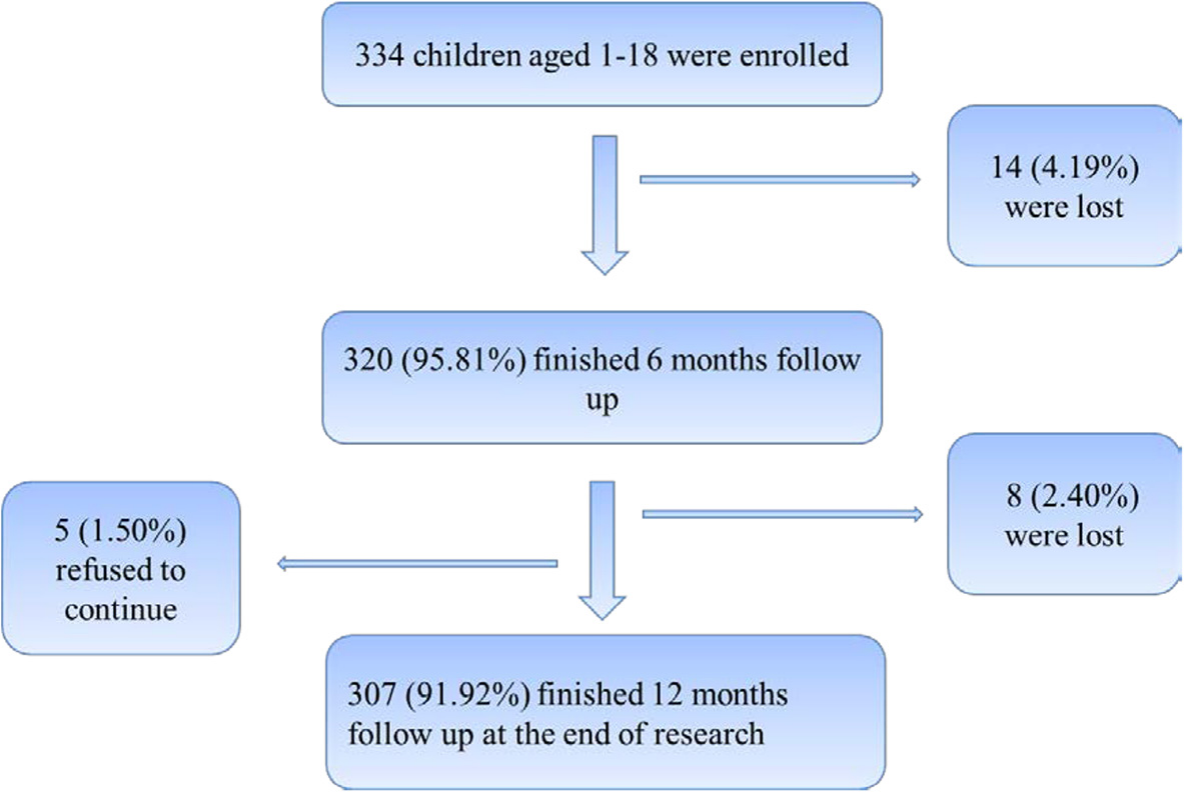
Participants recruitment and follow-up

Convenience sample recruiting was developed and we designed the sample size as 167 participants for each group. 334 children were enrolled with approximately 20 % lost-follow-up concerning in practical research, and all of them were followed up for 12 months prospectively. 307 (91.92 %) out of 334 completed follow-up and 27 (8.08 %) children dropped out; 22 (6.59 %) of them were lost and 5 (1.56 %) of them refused to continue (see Fig. 1).

11 (3.58 %) children did not suffer from RTI during 12-month follow-up period. 296 (96.42 %) children suffered at least 1 RTI episode within this period of time, 15 RTIs episodes was the most observed in this study. 4.32 (95 % CI 4.03-4.61) RTI episodes for each child in 12 months was the average.

### Characteristics of participants in both groups

The group with GHRS and group without GHRS were distinguished by GHRS scale identification. 160 (52.12 %) children with GHRS and 147 (47.88 %) without GHRS were in the observed cohorts.

Table 2 shows the characteristics of the groups and the baseline information from those groups.

**Table 2:** Characteristics of group with GHRS and group without GHRS

Characteristics	Group with GHRS (*N* = 160) n(%)/mean(±SD)	Group without GHRS (*N* = 147) n(%)/mean(±SD)	χ^2^/Z	*P* value
Gender			χ^2^ = 0.164	0.685
Female	66(41.25 %)	64(43.54 %)		
Male	94(58.75 %)	83(56.46 %)		
Ethnic group			χ^2^ = 1.260	0.262
Ethnic Han	146(91.25 %)	139(94.56 %)		
Other ethnic groups	14(8.75 %)	8(5.44 %)		
Character			χ^2^ = 0.796	0.672
Extrovert personality	113(70.63 %)	100(71.43 %)		
Moderate	35(21.88 %)	38(27.14 %)		
Introvert personality	12(7.50 %)	9(6.43 %)		
Vaccination against pneumonia			χ^2^ = 9.213	0.010
Yes	23(14.38 %)	9(6.12 %)		
No	50(31.25 %)	72(48.98 %)		
Not clear	87(54.37 %)	66(44.90 %)		
Vaccination against flu			χ^2^ = 2.942	0.230
Yes	15(9.38 %)	9(6.12 %)		
No	63(39.37 %)	67(45.58 %)		
Not clear	82(51.25 %)	71(48.30 %)		
Allergic history			χ^2^ = 1.760	0.185
Yes	27(16.88 %)	17(12.14 %)		
No	133(83.12 %)	130(87.86 %)		
Age at enrollment (years)	4.87(±2.15)	5.02(±2.36)	Z = −0.759	0.448
Height at enrollment (meters)	1.11(±0.14)	1.11(±0.16)	Z = −0.043	0.966
Weight at enrollment (kilograms)	20.68(±8.08)	19.74(±7.83)	Z = −1.651	0.099
BMI at enrollment (kg/m^2^)	16.39(±2.54)	15.55(±2.05)	Z = −3.079	0.002*

*P < 0.05 shows there is significant difference between two groups

It shows that there was no difference (*P* > 0.05) in gender, age, height, weight, ethnic group, character, vaccination against pneumonia, vaccination against flu, and allergic history between two groups, and there was difference (*P* < 0.05) in BMI between two groups.

### Occurrence of RTIs and RRTIs in both groups

Table 3 shows the specific episodes of RTIs during the follow-up visit, and Wilcoxon test were used to compare the RTI episodes between two groups.

**Table 3:** RTI episodes in two groups during follow-up visit

Follow-up visit period	Disease diagnosis		Group with GHRS	Group without GHRS	Z value	*P* value
3 months	URTI		232	179	−2.478	0.013*
	LRTI	Bronchitis	16	9	−0.471	0.638
		Pneumonia	2	2		
	RTI		250	190	−2.551	0.011*
6 months	URTI		449	329	−3.542	0.000**
	LRTI	Bronchitis	29	19	−0.553	0.581
		Pneumonia	6	7		
	RTI		484	355	−3.582	0.000**
9 months	URTI		606	441	−3.990	0.000**
	LRTI	Bronchitis	32	28	−0.319	0.750
		Pneumonia	8	8		
	RTI		646	477	−3.710	0.000**
12 months	URTI		723	519	−3.915	0.000**
	LRTI	Bronchitis	35	34	−0.240	0.811
		Pneumonia	9	9		
	RTI		767	562	−3.553	0.000**

*P < 0.05 shows there is significant difference between two groups

**P < 0.01 shows there is highly significant difference between two groups

Through Wilcoxon test, Z values of RTI episodes in 12 month between the two groups was −3.553.There was significant difference of RTI occurrence between group with GHRS and group without GHRS (*P* = 0.000).

The morbidity of RRTI in overall cohort was 38.11 %. 69 (43.13 %) children in the group with GHRS suffered from RRTI; there were 48 (32.65 %) children in group without GHRS. The risk ratio (RR) value of RRTI occurrence was 1.32 (95 % CI 0.91–1.91, *P* = 0.139), and the attributable risk percent (AR%) was 24.28 %.

### Bivariate analysis: linear correlation model

We built linear correlation models to explore the association between GHRS and RTI (URTI and LRTI) separately. It revealed that GHRS has a linear correlation with RTI over 12 months follow-up (Spearman correlation coefficient = 0.202, *P* = 0.000). GHRS has a linear correlation with URTI over 12 months follow-up (Spearman correlation coefficient = 0.225, *P* = 0.000).

We built linear correlation models to explore correlation between baseline factors, TCM symptoms and RTI episodes. Age (Spearman correlation coefficient = −0.142, *P* = 0.013), BMI (Spearman correlation coefficient = −0.143, *P* = 0.012), feverish feeling in palms and soles (Spearman correlation coefficient = 0.165, *P* = 0.004), irritability (Spearman correlation coefficient = 0.117, *P* = 0.041), sleep-talking (Spearman correlation coefficient = 0.142, *P* = 0.013), dry stool (Spearman correlation coefficient = 0.258, *P* = 0.000) and constipation (Spearman correlation coefficient = 0.166, *P* = 0.003) have a linear correlation with RTI episodes.

### Multivariate analysis

#### Linear regression model

Multicollinearity between independent variables were checked and not found to be present. Factors had been selected with P value less than 0.05 in our correlation model, and multiple linear regression models had been built in terms of those factors. In addition, a step-by-step elimination technique (α_in_ = 0.05,α_out_ = 0.10) was used to identify the independent variables.

At 12 months follow-up visit, dry stool, age, BMI and irritability were the major variables in the optimal model (Adjusted *R*^2^ = 0.114), with F value was 10.820 (*P* = 0.000) in the ANOVA test of the model, the linear regression model equation was y = 0.246x1 – 0.128x2 – 0.146x3 + 0.120x4 (y is RTI episodes in 12 months, x1 is dry stool, x2 is age, x3 is BMI, x4 is irritability) (see Table 4).

**Table 4:** Associated factors selected in linear regression model

	Unstandardiazed coefficients		Standardiazed coefficients	t value	*P* value
	Beta	Std. error	Beta		
Constant	6.361	1.002		6.347	0.000
Dry stool	0.608	0.133	0.246	4.569	0.000
Age	−0.147	0.064	−0.128	−2.296	0.022
BMI	−0.160	0.061	−0.146	−2.643	0.009
Irritability	0.299	0.135	0.120	2.211	0.028

The results showed that dry stool, BMI, age, irritability were independent variables for RTI occurrence over 12 months follow-up. Dry stool (*P* = 0.000) and irritability (*P* = 0.028) were positively correlated with RTI episodes, and age (*P* = 0.022) and BMI (*P* = 0.009) were negatively correlated with RTI episodes.

#### Logistic regression model

Multicollinearity between independent variables were checked and not found to be present. We built logistic regression model with these factors selected from correlation model with P value less than 0.2 to explore association among them and RRTI occurrence, and we used retrospective elimination techniques (α_in_ = 0.10, α_out_ = 0.15) to identify the independent variables.

Dry stool, age, BMI had been selected as independent variables in the optimal model, the logistic regression model equation was logit(P) = 2.038 + 0.412x1 – 0.117x2 – 0.153x3 (x1 is dry stool, x2 is age, x3 is BMI). The OR value of dry stool was 1.510 (95 % CI 1.200–1.900), the OR value of age was 0.858 (95 % CI 0.776–1.019), the OR value of BMI was 0.858 (95 % CI 0.763–0.965) (see Table 5).

**Table 5:** Associated factors screened in logistic regression model

	B	*P* value	OR value	95 % CI of OR value	
				Lower	Upper
Dry stool	0.412	0.000	1.510	1.200	1.900
Age	−0.117	0.091	0.889	0.776	1.019
BMI	−0.153	0.011	0.858	0.763	0.965
Constant	2.038	0.038	7.676		

The results showed that dry stool (*P* = 0.000) was positively correlated with RRTI occurrence, age (*P* = 0.091) and BMI (*P* = 0.011) were negatively correlated with RRTI occurrence.

## Discussion

The results suggested that more RTI episodes were reported in the GHRS group than the group without GHRS significantly (*P* < 0.05). It showed there might be positive association between GHRS and RRTI. As GHRS was a predictive factor, we could not deduce the cause-effect association between GHRS and RRTI. However, the association between GHRS and RRTI could be implied according to TCM theory [13].

Confounding factors were controlled in this study. All participants were aged from 1 to 18 without other underlying disease. The TCM diagnosis of participants were made by doctors with over 20 years clinical TCM experience. This research was conducted in unblinded real-world medical settings and all the data were acquired in clinical environment. The doctors who made TCM diagnosis did not take part in follow-up visits, and researchers who collected medical records in follow-up visits did not know exactly which group the participant belonged to. Therefore, blinding method was used in the data collecting and analyzing processes. Furthermore, there might be data missing during the follow up caused by recall bias in the cohort study, even though we collected data every 3 months, and participant booklets and medical record books were collected to compensate, which were used to record the sufferings and treatment in very detail by participants’ parents and the practitioners. There may be some bias caused by different investigators scoring the GHRS scale.

Two models had been built in this study to explore independent factors which were associated with RRTI. Dry stool was positively associated with RRTI, while age and BMI were negatively associated with RRTI. The results showed that there was a positive correlation between the extent of dry stool and RTIs in children, and extension of defecation time interval was positively correlated with RTIs as well. This study also suggested that BMI was negatively correlated with RTIs. Low BMI might due to prolonged GHRS which could impact digestive system function. TCM practitioners have realized that younger children are more prone to catching GHRS, especially the kindergarten children who are aged from 3 to 6 years old [13]. Therefore, it is necessary to explore how these factors including age, diet, BMI may contribute to the development of GHRS in the further research.

The findings of this prospective cohort study will be meaningful for TCM practitioners when treating RRTI children with GHRS. We have been suggesting that GHRS may be an important internal cause to catch RTIs, especially for children, for living standards have been greatly improved in China, and there are more food choices which are available for children. An unbalanced diet, such as rich in sugar, protein, energy, salt or additives, all above are difficult to be digested and absorbed effectively for children, which may lead to a GHRS thus predisposing to RRTI. Furthermore, GHRS may be ignored by practitioners and parents, while which is supposed to be considered as a predisposing factor.

TCM theories may help us understand the aetiology of chronic illness. This is a prospective cohort study that identified an association between GHRS and RRTI that should be tested prospectively. And a Grounded Theory study has been conducted to induce and generalize a theory by concerning and processing raw data from the perspectives of TCM practitioners’ understandings and experiences towards the interaction between GHRS and RRTI to theorize the results of this cohort study. Studies with larger sample size and longer follow up are needed in the future.

## Conclusions

There was association between GHRS and RRTI. We found that dry stool and BMI were the factors which impacted most, and dry stool was positively associated with RRTI, BMI was negatively associated with RRTI. Studies with larger sample size and longer follow up are needed in the future to further evaluate this association. Relieving GHRS symptoms should be considered when practitioners treating RRTI children with GHRS.

## Abbreviations

AR%: attributable risk percent
ARTI: acute respiratory tract infection
AURI: acute upper respiratory infection
BMI: body max index
CI: confidence intervals
CRF: case report form
FM1: A/Fort Monmouth/1/47(H1N1)
GHRS: gastrointestinal heat retention syndrome
LRTI: lower respiratory tract infection
OR: odds ratios
RR: relative risk
RRTI: recurrent respiratory tract infection
RTI: respiratory tract infection
SOP: standard operating procedure
TCM: Traditional Chinese Medicine
URTI: upper respiratory tract infection
